# Co-infection of Cytomegalovirus and Epstein-Barr Virus Diminishes the Frequency of CD56^dim^NKG2A^+^KIR^−^ NK Cells and Contributes to Suboptimal Control of EBV in Immunosuppressed Children With Post-transplant Lymphoproliferative Disorder

**DOI:** 10.3389/fimmu.2020.01231

**Published:** 2020-06-17

**Authors:** Janice K. P. Lam, Tarik Azzi, K. F. Hui, Aikha M. G. Wong, Donal McHugh, Nicole Caduff, K. H. Chan, Christian Münz, Alan K. S. Chiang

**Affiliations:** ^1^Department of Paediatrics and Adolescent Medicine, Li Ka Shing Faculty of Medicine, Queen Mary Hospital, The University of Hong Kong, Pok Fu Lam, Hong Kong; ^2^Experimental Infectious Diseases and Cancer Research, University Children's Hospital of Zurich, Zurich, Switzerland; ^3^Department of Viral Immunobiology, Institute of Experimental Immunology, University of Zurich, Zurich, Switzerland; ^4^Department of Microbiology, Li Ka Shing Faculty of Medicine, Queen Mary Hospital, The University of Hong Kong, Hong Kong, China

**Keywords:** EBV, post-transplant lymphoproliferative disorder, infectious mononucleosis, natural killer cells, NKG2A, KIR, immunosuppression, cytomegalovirus

## Abstract

Post-transplant lymphoproliferative disorder (PTLD) is a rare but potentially life-threatening complication, frequently associated with Epstein-Barr virus (EBV), which develops after solid organ or stem cell transplantation. Immunosuppression received by transplant recipients has a significant impact on the development of PTLD by suppressing the function of T cells. The preferential proliferation of NKG2A-positive natural killer (NK) cells during primary symptomatic EBV infection known as infectious mononucleosis (IM) and their reactivity toward EBV-infected B cells point to a role of NK cell in the immune control of EBV. However, NK cell-mediated immune response to EBV in immunosuppressed transplant recipients who develop PTLD remains unclear. In this study, we longitudinally analyzed the phenotype and function of different NK cell subsets in a cohort of pediatric liver transplant patients who develop PTLD and compared them to those of children with IM. We found persistently elevated plasma EBV DNA levels in the PTLD patients indicating suboptimal anti-viral immune control. PTLD patients had markedly decreased frequency of CD56^dim^NKG2A^+^Killer Immunoglobulin-like receptor (KIR)^−^ NK cells from the time of diagnosis through remission compared to those of IM patients. Whilst the proliferation of CD56^dim^NKG2A^+^KIR^−^ NK cells was diminished in PTLD patients, this NK cell subset maintained its ability to potently degranulate against EBV-infected B cells. Compared to cytomegalovirus (CMV)-seropositive and -negative IM patients, PTLD patients co-infected with CMV and EBV had significantly higher levels of a CMV-associated CD56^dim^NKG2C^hi^CD57^+^NKG2A^−^KIR^+^ NK cell subset accumulating at the expense of NKG2A^+^KIR^−^ NK cells. Taken together, our data indicate that co-infection of CMV and EBV diminishes the frequency of CD56^dim^NKG2A^+^KIR^−^ NK cells and contributes to suboptimal control of EBV in immunosuppressed children with PTLD.

## Introduction

Epstein-Barr Virus (EBV) is a herpesvirus that latently infects more than 90% of the world's population (1). It is renowned for its potent B-cell transforming capability to drive numerous benign and malignant lymphoproliferative disorders such as infectious mononucleosis (IM), post-transplant lymphoproliferative disorder (PTLD), and Burkitt lymphoma (2). Contact with saliva is known to be the major mode of transmission of EBV, whereas solid organ or stem cell transplantation and rarely blood transfusion can be alternative routes to transmit EBV. Primary infection of EBV is usually asymptomatic and occurs in early childhood, yet, delayed infection can develop into IM.

IM is a self-limiting disorder characterized by extensive proliferation of polyclonal EBV-specific CD8^+^ T cells in response to primary EBV infection (3–5). Extensive work has been performed to report the suppressive effect of T cells on EBV-infected cells and our group has previously shown that the emergence of polyfunctional CD4^+^ and CD8^+^ T cells plays a role in viral control during IM (6). Besides T cells, the antiviral role of natural killer (NK) cells in EBV-specific immune control has also received attention in recent decades. Individuals with selective primary NK cell deficiency exhibit an increased susceptibility to EBV, associated with fatal primary infection or the development of EBV-associated cancers (7, 8). In addition, depletion of NK cells upon EBV infection of mice harboring humanized immune system components is associated with higher EBV DNA levels and points to a non-redundant antiviral role of these immune cells (4). Furthermore, the preferential proliferation and accumulation of early-differentiated CD56^dim^NKG2A^+^KIR^−^ NK cells in immunocompetent children with acute IM indicate that the NK cell compartment reacts specifically to EBV (5). This is also highlighted by the fact that other viral infections are associated with the accumulation of another NK cell subset, such as the late-differentiated CD56^dim^NKG2C^hi^CD57^+^ NK cell subset upon human cytomegalovirus (CMV) infection (9). Finally, the demonstration of efficient *in vitro* killing of autologous EBV-infected lymphoblastoid cell lines (LCL) by NKG2A^+^ NK cells (10) indicates a potential role of NK cells in the immune control of EBV-associated B cell cancers. The study of IM patients provides insight in the pathogenesis and immune control in primary EBV infection.

Compared to IM, less is known about EBV-specific immune control in PTLD. PTLD is a rare but life-threatening complication frequently associated with EBV which develops after solid organ or stem cell transplantation (11, 12). The incidence of PTLD is around 1–20% of solid organ transplant recipients and depends on the transplanted organ, the degree of immunosuppression and the serological EBV donor-recipient constellation. For instance, adults who underwent liver transplantation have an incidence of PTLD of around 1–4.3% (13, 14). However, in pediatric liver transplant recipients, the incidence of PTLD is 6% in the first year, and reaches a 5-year cumulative incidence rate of 20% (Chiang AKS, unpublished data). The immunosuppressive regimen and the EBV seronegative constellation of the solid organ transplant (SOT) recipient frequently encountered in pediatric transplantation are independent risk factors for the development of PTLD (15). The impact of immunosuppression on T cell functions has been reported extensively. Vafadari et al. (16) reported that tacrolimus suppressed the T cell activation via the NF-_K_B pathway. In line with this finding, Jones et al. (17) also reported that the frequencies of EBNA1- and BZLF1-specific CD4^+^ interferon gamma (IFNγ)-producing T cells were decreased in PTLD patients compared to healthy individuals. Another study reported increased PD-1 expression on CD8^+^ T cells in transplant recipients with EBV infection suggesting T cell exhaustion (18), although PD-1^+^ CD8^+^ T cells retained protective functions in humanized mice infected with EBV (19).

NK cells work complementary to T cells in controlling tumors and viral infections. Their adaptive immune features exhibited during viral infection in experimental mice (20) and proliferation in humans during IM provide evidence that NK cells also play a role in controlling EBV infection (5). NK cells in the peripheral blood are composed of two main subsets, i.e., CD56^bright^CD16^−^ NK cells and CD56^dim^CD16^+^ NK cells that differ in terms of phenotype and function (21). NK cells express activating receptors such as NKp46, NKp30, and NKG2D and inhibitory receptors such as NKG2A and KIRs. The balance between activating and inhibitory signals upon recognition of virus-infected cells determines the NK cell response (21, 22). To date, some NK studies in PTLD patients have characterized the phenotype of NK cells, for example, Wiesmayr et al. (23) reported a reduction of NKp46 and NKG2D surface expression level in pediatric PTLD patients compared to healthy children and pediatric SOT recipients without PTLD. However, the contribution of distinct NK cell subsets to the immune control of EBV and the longitudinal development of NK cell subsets in PTLD patients remain largely unknown.

In the present study, we longitudinally assessed the phenotype and function of NK cell subsets in the peripheral blood of immunosuppressed pediatric liver transplant patients who developed PTLD and compared them with those of immunocompetent pediatric patients who developed IM.

## Materials and Methods

### Subject Recruitment

Fifteen pediatric liver transplant recipients diagnosed with PTLD under 18 years of age were recruited and followed longitudinally. The primary disease was biliary atresia (*n* = 15). Long term immunosuppressive regimen of the PTLD patients consisted mainly of the calcineurin inhibitor, tacrolimus. All patients included in this study were diagnosed to have PTLD based on either histological examination of biopsy tissues (*n* = 14) or radiological examination using PET-CT scan (*n* = 1; Supplementary Table 1). EBV serological status prior to liver transplantation was obtained (*n* = 11). Quantification of EBV copies and serological screening for EBV were performed to confirm the infection status at diagnosis of PTLD (Table 1). PTLD patients who tested positive or negative for EBV viral capsid antigen (VCA) IgM and positive for VCA IgG but negative for Epstein-Barr nuclear antigen (EBNA) IgG were considered to have primary EBV infection. The majority of the patients had primary EBV infection at diagnosis of PTLD (*n* = 13). All PTLD patients showed EBV and CMV seroconversion. Detailed clinical characteristics of the PTLD patients are listed in Supplementary Table 1.

**Table 1:** Basic characteristics of 17 IM and 15 PTLD patients at diagnosis.

	**IM (*n* = 17)**	**PTLD (*n* = 15)**
Age at diagnosis (years)	3.57 ± 2.04	2.56 ± 1.16
Male	12	5
Presence of EBV VCA IgM	14	10
EBV VCA IgG titers	2192.9 ± 2299.1	26176 ± 41328.7
Presence of EBNA IgG by ACIF	0	2
EBV DNA levels at diagnosis (copies/mL plasma)	103536 ± 211995.9	855272.60 ± 1957002

*Mean ± standard deviation was shown. VCA, viral capsid antigen; EBNA, Epstein-Barr nuclear antigen; ACIF, anti-complement immunofluorescence*.

Seventeen pediatric acute IM patients were also recruited and followed longitudinally. Patients with typical IM symptoms, such as fever, sore throat and lymphadenopathy, who tested positive or negative for EBV VCA IgM and positive for VCA IgG but negative for EBNA IgG were enrolled in this study (Table 1). Twenty healthy adults were recruited and served as healthy controls.

Heparinized peripheral blood samples were collected at the following time points, diagnosis, around 1 month, 3 months, 6 months, and 12 months. Additionally, the sample collection of PTLD patients continued at yearly intervals post-diagnosis. If sampling was performed at time points varying from the above mentioned analysis time points, e.g., for clinical reasons, the sample closest to the missing time point was selected. The mean time post-diagnosis and the standard deviation between the proposed and the actual time point selected are listed for all PTLD and IM subjects in Supplementary Table 2.

Plasma was isolated from each individual and stored at −80°C until use. Peripheral blood mononuclear cells (PBMCs) were isolated by standard Ficoll-Hypaque density gradient centrifugation. Collected PBMCs were cryopreserved in fetal bovine serum (FBS) (Invitrogen, CA) with 10% DMSO in liquid nitrogen until use.

All patient samples were handled as potential biohazardous materials following the institutional safety procedures. The study protocols were approved by the Institutional Review Board of The University of Hong Kong. Informed written consent was obtained from each participant prior to the study in accordance with the Declaration of Helsinki.

### DNA Extraction and Quantification of EBV Copies

The NucliSENS easyMag instrument (BioMerieux) was used to extract DNA from plasma in accordance to the manufacturer's instruction. Plasma EBV DNA levels were determined by detecting the EBV BamH1_W repeats in EBV genomes by quantitative PCR (qPCR) with ABI PRISM 7900 sequence detector (Applied Biosystems). The forward and reverse primers with the following sequences were used: 5′-GGTCGCCCAGTCCTACCA-3′ and 5′-GCTTACCACCTCCTCTTCTTGCT-3′, respectively. The FAM-labeled probe consisted of the following sequence: 5′-CCAAGAACCCAGACGAGTCCGTAGAAGG-3′. Standard curve for copy numbers were obtained using serial dilutions of Namalwa DNA. All samples were performed in triplicate and the mean results were reported as EBV copies per mL plasma.

### Analysis of NK Cell Subset Phenotype and Proliferation

Frozen PBMCs were thawed, washed and stained with the following combination of monoclonal antibodies, Pacific Blue-conjugated anti-CD3 (Biolegend), PE-Cy7-conjugated anti-CD56 (BD pharmingen), APC-Cy7-conjugated anti-CD16, PE-conjugated anti-NKG2A (Beckman Coulter), APC-conjugated anti-CD158a/h (Biolegend), APC-conjugated anti-CD158b1/b2/j (Biolegend), APC-conjugated anti-KIR3DL1 (Biolegend), Alexa Fluor 488-conjugated anti-NKG2C (R&D Systems), APC-Cy7-conjugated anti-CD27 (BD pharmingen), and PerCP-Cy5.5 conjugated anti-CD57 (Biolegend) monoclonal antibody. LIVE/DEAD Fixable Aqua was used for dead cell exclusion in every phenotypic analysis. The stained cells were then washed, fixed and permeabilized by BD Cytofix/Cytoperm Fixation/Permeabilization kit (BD Biosciences) according to the manufacturer's protocol. Subsequently, cells were washed and stained intracellularly with FITC-conjugated anti-Ki67 (BD Pharmingen) for 30 min at 4°C. Cells were washed and re-suspended in PBS prior to flow cytometric analysis. Cells were acquired on FACS LSR-II and LSR Fortessa (BD Biosciences) and FACS data was analyzed by FlowJo v10 (BD). Samples with <20% live lymphocytes were excluded from the analysis.

### Stimulation of NK Cells

Frozen PBMCs were thawed and rested overnight in 10% FBS/RPMI 1640 with stimulation of IL-2 (100 U/mL). PBMCs (5 × 10^5^) were subsequently co-cultured with LCL721.221 or K562 at an effector-to-target ratio of 10:1 for 6 h at 37°C, 5% CO_2_ in the presence of Pacific Blue-conjugated anti-CD107a (Biolegend). After 1 h of incubation, the protein transport inhibitor Monensin (1 μg/ml BD Golgi Stop, BD Pharmingen) was added to all samples. PBMCs only and PBMCs stimulated with PMA (5 ng/mL) and Ionomycin (500 ng/mL) were used as negative and positive controls, respectively.

### Flow Cytometry Based Degranulation Assay

After incubation for 6 h, cells were stained with the following combination of monoclonal antibodies, FITC-conjugated anti-CD3 (Biolegend), PE-Cy7-conjugated anti-CD56 (BD pharmingen), PE-conjugated anti-NKG2A (Beckman Coulter), APC-conjugated anti-CD158a/h (Biolegend), APC-conjugated anti-CD158b1/b2/j (Biolegend), and APC-conjugated anti-KIR3DL1 (Biolegend) monoclonal antibody for surface marker staining. LIVE/DEAD Fixable Aqua (Invitrogen) was stained for dead cell exclusion in each sample. The stained cells were subsequently washed, fixed, and permeabilized by BD Cytofix/Cytoperm Fixation/Permeabilization kit (BD Biosciences) according to the manufacturer's protocol. Cells were washed and re-suspended in PBS prior to flow cytometric analysis. Cells were acquired on FACS LSR-II flow cytometer (BD Biosciences) and FACS data were analyzed by FlowJo v10 (BD). Samples with <20% live lymphocytes were excluded from the analysis. The spontaneous degranulation signals from PBMCs only were subtracted in each analysis.

### Cell Lines

LCL721.221 and K562 were maintained in 10% fetal bovine serum (FBS)/RPMI 1640. Both cell lines tested negative for mycoplasma contamination.

### Statistical Analysis

Wilcoxon matched-pairs signed rank tests and Mann-Whitney tests were applied to compare data derived from one group or those between groups, respectively. Correlation between plasma EBV DNA levels and blood tacrolimus levels in pediatric PTLD patients was determined by Spearman test. A *p* < 0.05 was regarded as statistically significant. Prism 6 (GraphPad Software, La Jolla, CA) was used for calculations and illustrations.

## Results

### PTLD Patients Exhibit Persistently Elevated Plasma EBV DNA Levels Up to 24 Months After Diagnosis

**Figure 1:**
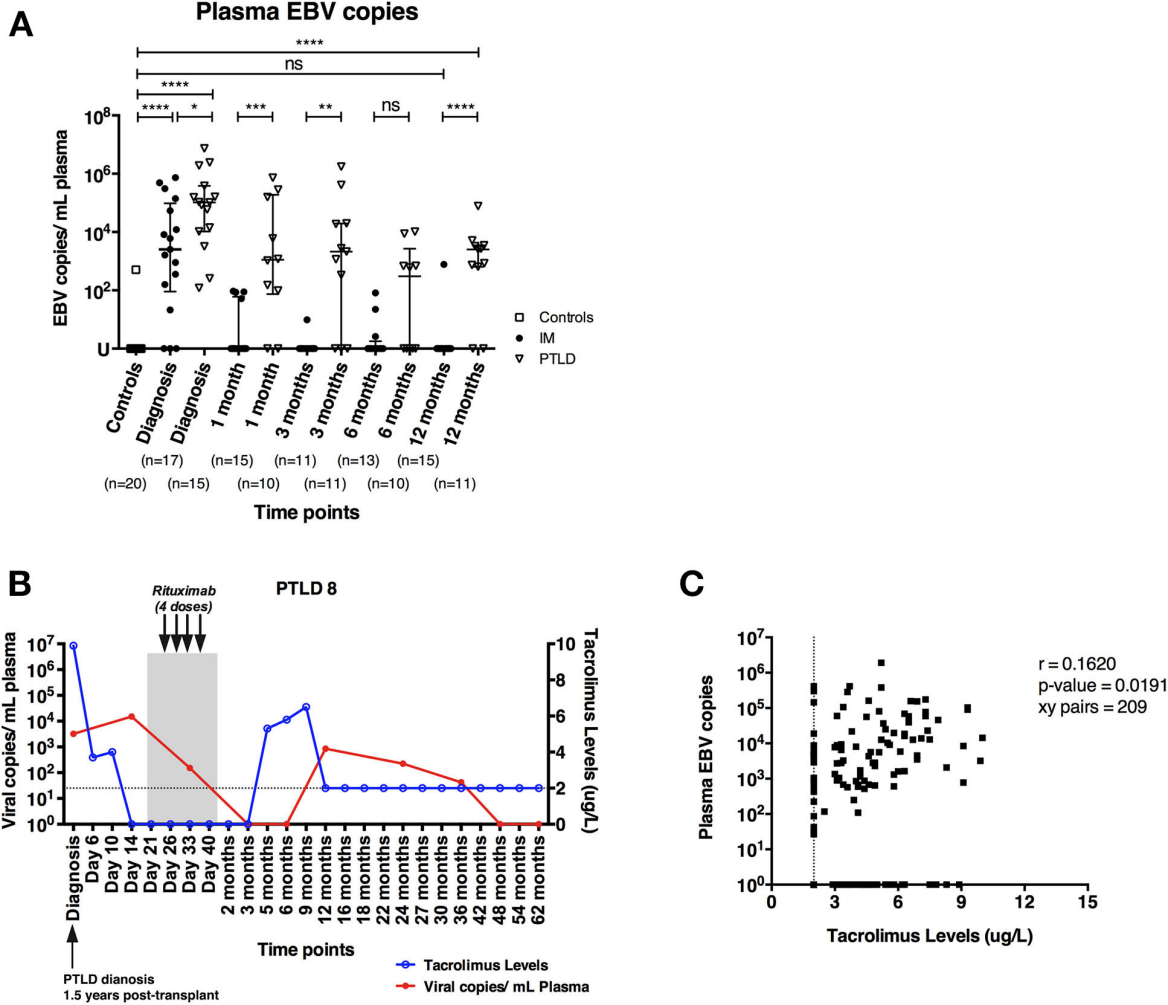
PTLD patients exhibit persistently elevated plasma EBV DNA levels up to 12 months after diagnosis and plasma EBV DNA levels show weak correlation with blood tacrolimus levels. **(A)** Plasma EBV DNA levels in IM patients and PTLD patients were assessed at diagnosis, as well as during recovery at 1, 3, 6, and 12 months post-diagnosis. Twenty healthy adults were used as healthy controls for detecting baseline EBV DNA levels. **(B)** Kinetics of plasma EBV DNA levels and blood tacrolimus levels in 1 representative PTLD patient is plotted from diagnosis (D0) to longitudinal time points. Blood tacrolimus levels ≤2 μg/L were not detectable and are plotted on the dotted line depicting the detection limit. The date of PTLD diagnosis, the period and treatment of each PTLD patient (gray area) are also plotted. The plasma EBV DNA levels (red line) are plotted on left y-axis, while the blood tacrolimus levels (blue line) are plotted on right y-axis. **(C)** Relationship between plasma EBV DNA levels and the blood tacrolimus levels in 13 PTLD patients at various time points ranging from diagnosis to their latest follow-up time points. Two patients were excluded due to the lack of measurable pairs of tacrolimus levels and plasma EBV DNA levels for the analysis. EBV DNA levels were quantified by qPCR and reported as EBV copy numbers per ml plasma. Median ± interquartile range is shown. Mann-Whitney tests were applied to compare the plasma EBV DNA levels in different cohorts of patients at all time points. Two-tailed spearman test was applied to calculate the correlation between plasma EBV DNA levels and blood tacrolimus in PTLD patients. U, undetectable level; dotted line, limit of detection of tacrolimus in blood; ns, *p* > 0.05; *p ≤ 0.05; **p ≤ 0.01; ***p ≤ 0.001; and ****p ≤ 0.0001.

EBV DNA levels in the plasma of 17 pediatric IM patients and 15 pediatric liver transplant patients with PTLD were quantified longitudinally via qPCR starting at the time point of diagnosis until 12 and 24 months later, respectively. In addition, we assessed plasma EBV DNA levels of 20 healthy adults, amongst whom only one had plasma viremia above the detection threshold. In contrast, we found elevated EBV DNA levels in the plasma of both IM and PTLD patients at diagnosis, yet levels in PTLD patients were significantly higher (Figure 1A). The median plasma EBV DNA levels decreased in both cohorts starting 1 month after diagnosis. Although EBV DNA levels were below the detection limit in most of the IM patients at 3 months after diagnosis, EBV DNA levels remained elevated in more than half of the PTLD patients during the follow-up and only decreased slowly over time. At 12 months post-diagnosis, plasma EBV viremia was found in 82% of PTLD patients (9 out of 11) compared to 7% of IM patients (1 out of 15). Even 2 years post-diagnosis, 50% of PTLD patients had detectable EBV DNA levels in the plasma (Supplementary Figure 1). These results suggest that EBV DNA levels tend to persist at elevated levels in PTLD patients after clinical recovery.

### Plasma EBV DNA and Blood Tacrolimus Levels Correlate Weakly in PTLD Patients

Previous studies reported that a subgroup of immunosuppressed solid organ transplant recipients exhibited subclinical high EBV DNA levels after transplantation without developing EBV-associated PTLD (24, 25). In some of these patients, EBV viremia persisted for several months and a link with the immunosuppressive treatment was postulated. Therefore, we assessed the kinetic changes of plasma EBV DNA and trough blood tacrolimus levels in 15 PTLD patients individually from the time of diagnosis through longitudinal time points.

We found that patients with increased blood tacrolimus levels had increased plasma EBV DNA levels (Figure 1B). In general, most of the patients had lower tacrolimus levels and exhibited decrease in plasma EBV DNA levels during recovery, likely owing to the reductions in immunosuppressive treatment following diagnosis of PTLD. Nevertheless, a weak positive correlation between blood tacrolimus and plasma EBV DNA levels was found in PTLD patients (*r* = 0.1620; *p* = 0.0191; Figure 1C).

In line with previously published results on the effect of immunosuppression and EBV DNA levels on the development of PTLD (26, 27), these findings suggest that immunosuppression leads to suboptimal immune control of EBV as indicated by the persistently elevated plasma EBV DNA levels in the PTLD patients.

### The Frequency of CD56^dim^NKG2A^+^KIR^−^ NK Cells Is Significantly Higher in IM Than in PTLD Patients

**Figure 2:**
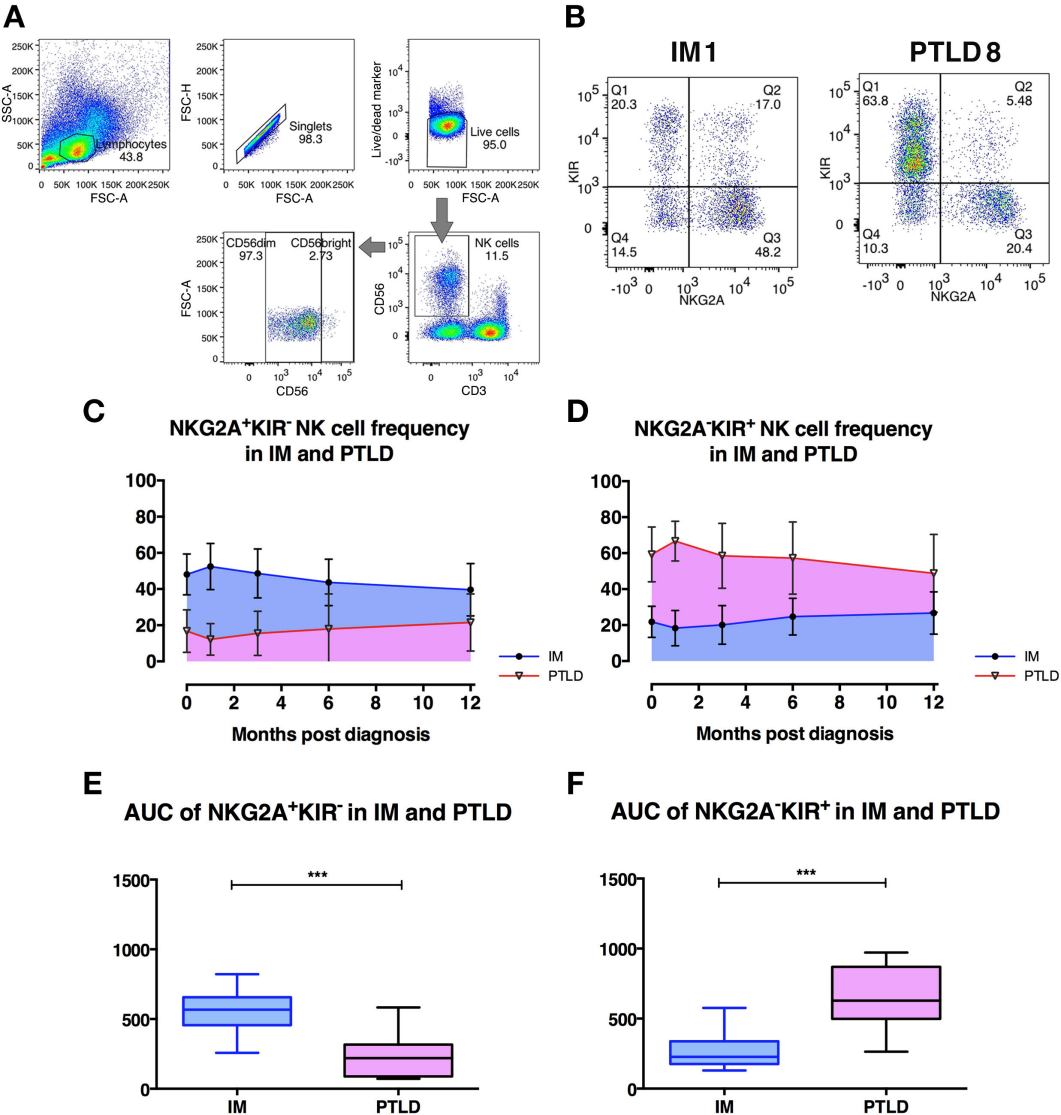
The frequency of CD56^dim^ NKG2A^+^KIR^−^ NK cells is significantly higher in IM than in PTLD patients. **(A)** Gating strategy for flow cytometric analysis of CD56^+^ NK cells in 1 representative PTLD patient at diagnosis. **(B)** Gating strategy for flow cytometric analysis of CD56^dim^NKG2A^−^KIR^+^ and CD56^dim^NKG2A^−^KIR^+^ NK cell subsets in representative IM and PTLD patients. **(C**, **D)** The mean ± SD showing **(C)** CD56^dim^NKG2A^+^KIR^−^ NK cell frequency over time and **(D)** CD56^dim^NKG2A^−^KIR^+^ NK cell frequency over time in 15 IM and 13 PTLD patients from diagnosis to 12 months post-diagnosis time point. **(E)** Median ± interquartile range and minimum and maximum values of area-under-the-curve (AUC) of CD56^dim^NKG2A^+^KIR^−^ NK cell frequency vs. time (from **C**) and **(F)** CD56^dim^NKG2A^−^KIR^+^ NK cell frequency vs. time (from **D**) between IM and PTLD patients compared via Mann-Whitney tests. ***p ≤ 0.001. Blue line, IM patients; Pink line, PTLD patients.

Immunosuppressive drugs administered to transplant recipients, such as tacrolimus, inhibit the function of T cells that could cause graft rejection, but increase the risk for PTLD (1, 2). Besides T cells, several studies have reported potential roles of NK cells in EBV control (4, 5). To identify NK cell subsets that could play a role in the immune control of EBV, we assessed the frequencies of different NK cell subsets in 17 IM and 15 PTLD patients at time points ranging from diagnosis (D0) to 12 or 24 months, respectively, via flow cytometric analysis (Figures 2A,B).

Overall frequencies of CD3^−^CD56^+^ NK cells did not differ between both cohorts of patients at different time points (Supplementary Figure 2A). In both cohorts, the CD56^dim^ NK cell subset represented more than 90% of the NK cells in peripheral blood as previously reported in healthy individuals (Supplementary Figure 2B). The median frequency of the CD56^bright^ NK cell subset also did not differ between IM and PTLD patients (data not shown). Interestingly, a significant decrease in the frequency of CD56^dim^ NK cell subset was observed in IM patients after 3 and 6 months, while in PTLD patients, it remained stable throughout the observation period.

Next, we assessed the distribution of subsets within CD56^dim^ NK cells according to the expression of the inhibitory receptors, NKG2A and KIRs. Strikingly, the majority of CD56^dim^ NK cells in IM patients was NKG2A^+^KIR^−^ (Figure 2C), while in PTLD patients, the majority was NKG2A^−^KIR^+^ (Figure 2D). Similar to PTLD patients, healthy controls showed a majority of NKG2A^−^KIR^+^ NK cell subset within CD56^dim^ NK cells (data not shown). Analysis of the subset frequencies within the first year post-diagnosis demonstrated significant differences between the two patient cohorts. The frequencies of CD56^dim^NKG2A^+^KIR^−^ NK cells over time were significantly higher in IM patients (Figure 2E), whereas in PTLD patients, NKG2A^−^KIR^+^ subset frequencies over time were significantly higher (Figure 2F). We then examined whether treatment administered to PTLD patients affected the frequencies of NKG2A^+^KIR^−^ and NKG2A^−^KIR^+^ NK cells. No significant difference was shown in the frequencies of NKG2A^+^KIR^−^ and NKG2A^−^KIR^+^ NK cells before and after treatment of PTLD patients (Supplementary Figures 2C,D). Thus, the observed quantitative deficiency of protective CD56^dim^NKG2A^+^KIR^−^ NK cells might contribute to suboptimal immune control of EBV associated with the development of PTLD.

### CD56^dim^NKG2A^+^KIR^−^ NK Cells Proliferate Less in PTLD Than in IM Patients

**Figure 3:**
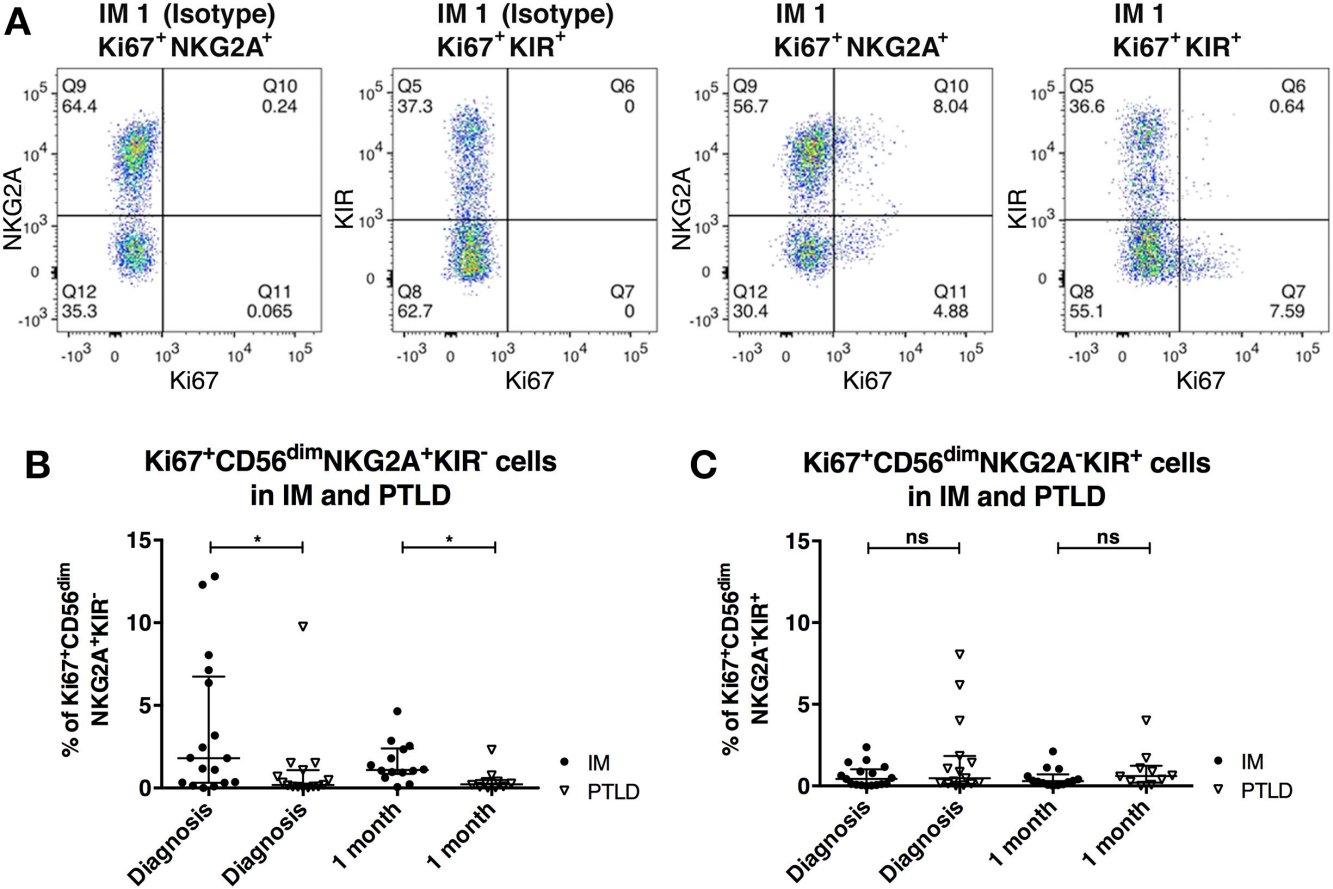
CD56^dim^NKG2A^+^KIR^−^ NK cells proliferate less in PTLD compared to those in IM patients shortly after diagnosis. **(A)** Flow cytometric gating of the frequency of Ki-67 expressing cells of CD56^dim^NKG2A^+^KIR^−^ and CD56^dim^NKG2A^−^KIR^+^ NK cell subsets with isotype control antibody or anti-human Ki67 antibody in 1 representative IM patient. Frequency change of Ki-67 expressing cells of **(B)** CD56^dim^NKG2A^+^KIR^−^ NK cell subset and **(C)** CD56^dim^NKG2A^−^KIR^+^ NK cell subsets in 17 IM and 15 PTLD patients at diagnosis and 1 month post-diagnosis, respectively. Median ± interquartile range is shown. Mann-Whitney tests were applied to compare different groups. ns, *p* > 0.05 and *p ≤ 0.05.

We next investigated whether differences in NK cell proliferation could explain the lower frequencies of CD56^dim^NKG2A^+^KIR^−^ NK cells in PTLD patients. To that end, we assessed the expression of the intranuclear proliferation marker Ki-67 within the different NK cell subsets (Figure 3). The frequency of Ki-67^+^ cells within the CD56^dim^NKG2A^+^KIR^−^ subset was significantly higher in IM than in PTLD patients during the acute phase of the disease (Figure 3B). Moreover, unlike in IM subjects, only a low frequency of NKG2A^+^KIR^−^ NK cells proliferated in PTLD patients during the observation period. In contrast, the levels of Ki-67 expressing cells among CD56^dim^NKG2A^−^KIR^+^ cells did not significantly differ between the two patient cohorts nor did they change over time (Figure 3C). Minimal proliferation of CD56^dim^NKG2A^+^KIR^−^ NK cells was detected in the healthy controls (data not shown).

The increased proliferation in CD56^dim^NKG2A^+^KIR^−^ NK cells compared to the CD56^dim^NKG2A^−^KIR^+^ NK cells in acute IM patients is in line with one previous study (5) and supports previous evidence for an important role of this subset in resolving this self-limiting disease. Furthermore, it suggests that a deficiency in this subset may contribute to the suboptimal viral immune control observed in PTLD.

### Degranulation of CD56^dim^NKG2A^+^KIR^−^ NK Cell Subset in Response to EBV-Infected B Cells Is Maintained in PTLD Patients

**Figure 4:**
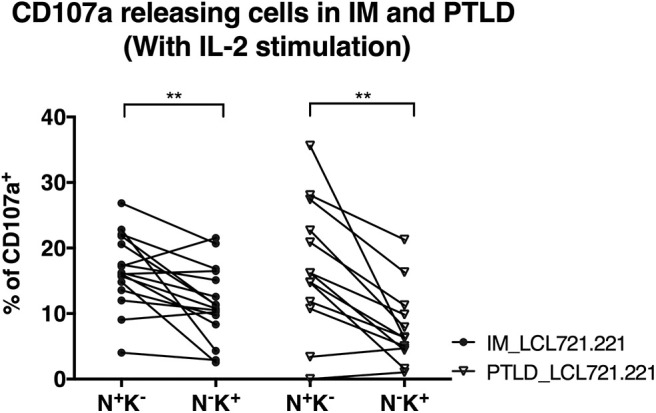
CD56^dim^NKG2A^+^KIR^−^ NK cells of both PTLD and IM patients exhibited comparable cytotoxic degranulation in response to EBV-infected B cells. PBMCs from 16 IM patients at the 12 months recovery time point and PBMCs from 13 PTLD patients at the 24 months recovery time point were stimulated with IL-2 overnight prior to co-culture with the HLA class I negative EBV-infected B cell line LCL721.221 on the next day at effector to target ratio of 10:1 for 6 h. Frequencies of cells with CD107a surface expression in the CD56^dim^NKG2A^+^KIR^−^ and CD56^dim^NKG2A^−^KIR^+^ NK cell subsets in 16 IM patients and 13 PTLD patients were assessed by flow cytometry at the end of the co-culture. Every line represents 1 individual. Wilcoxon matched-pairs signed rank tests were applied to compare data from one group. **p ≤ 0.01.

We performed degranulation assays to examine differences in the protective effects of NK cell subsets obtained from the two patient cohorts after clinical recovery. Upon contact with the HLA class I negative EBV-infected B cell line LCL721.221, the CD56^dim^NKG2A^+^KIR^−^ NK cell subset degranulated significantly more than the CD56^dim^NKG2A^−^KIR^+^ subset in both patient cohorts, as determined by CD107a staining (Figure 4). The same effect was observed against the HLA-I and II negative erythroleukemia cell line K562, which generally elicited a stronger response (Supplementary Figure 3). The similar cytotoxicity of CD56^dim^NKG2A^+^KIR^−^ NK cells from IM and PTLD patients argues against different NKG2A mediated licensing due to different HLA class I distribution in the two patient cohorts (28).

In addition, the degranulation potency of CD56^dim^NKG2A^+^KIR^−^ NK cell subsets in response to LCL721.221 (Figure 4) or K562 (Supplementary Figure 3) was not different between the two patient cohorts. The similar cytotoxicity of CD56^dim^NKG2A^−^KIR^+^ NK cells from IM and PTLD patients argues against different KIR mediated licensing due to different HLA class I distribution in the two patient cohorts (29). These findings suggest that the CD56^dim^NKG2A^+^KIR^−^ NK cell subset in PTLD patients is capable of efficient degranulation upon challenge by latently EBV-infected B cells and contributes to targeting EBV-infected B cells. The suboptimal EBV-specific immune control at later time points after diagnosis in PTLD patients correlates with the diminished frequency of potent degranulating CD56^dim^NKG2A^+^KIR^−^ NK cells compared to that of IM patients.

### Expansion of CD56^dim^NKG2C^hi^CD57^+^ NK Cells Is Associated With Diminished Frequency of CD56^dim^NKG2A^+^KIR^−^ NK Cells in PTLD Patients

**Figure 5:**
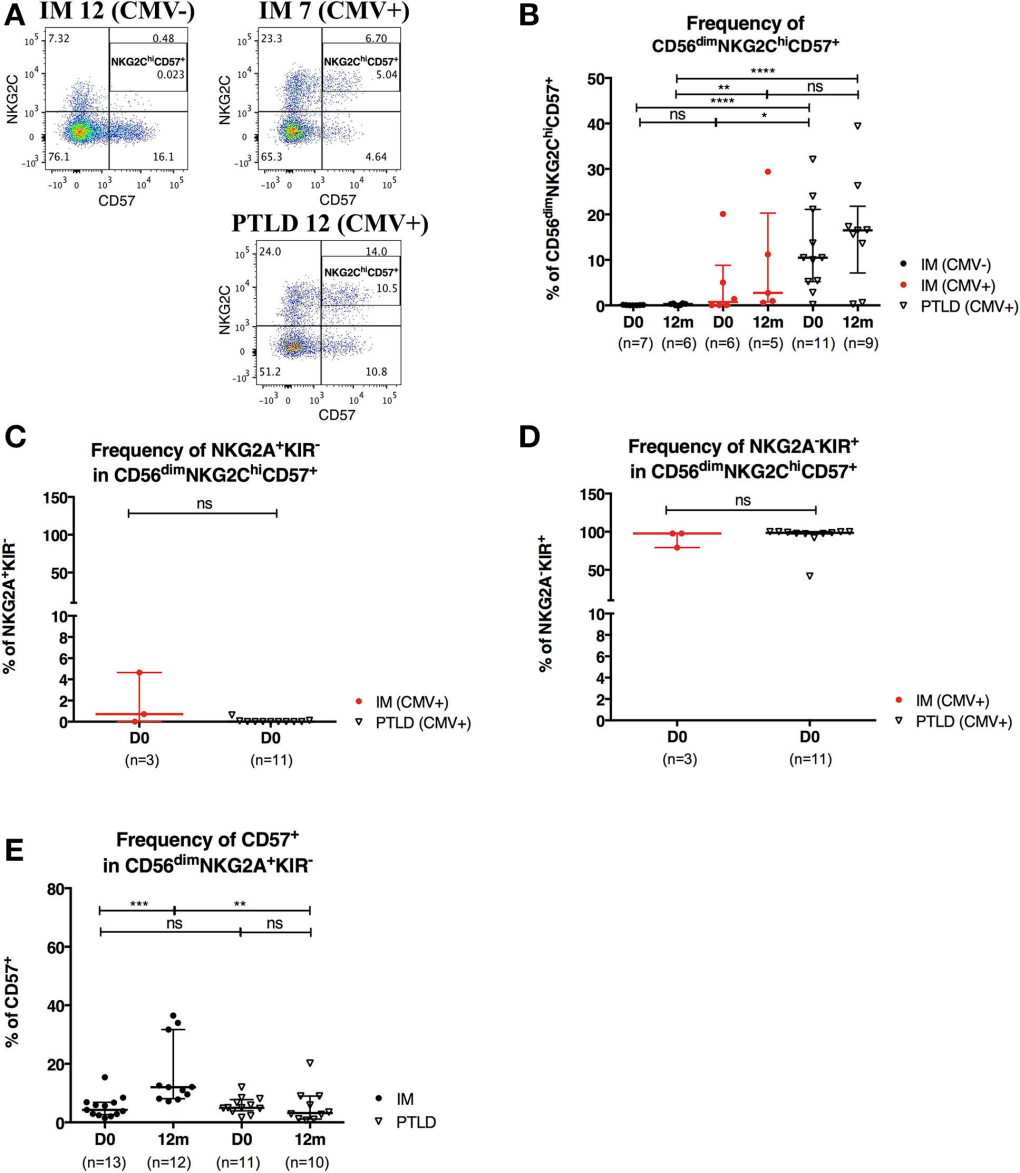
CD56^dim^NKG2C^hi^CD57^+^ NK cells are found in higher frequency in PTLD than in IM patients. **(A)** Gating strategy for flow cytometric analysis of CD56^dim^NKG2C^hi^CD57^+^ cells in representative CMV-seronegative (CMV–) IM, CMV-seropositive (CMV+) IM and PTLD patients at diagnosis. **(B)** Frequency of CD56^dim^NKG2C^hi^CD57^+^ NK cells in 7 CMV– IM, 6 CMV+ IM and 11 PTLD patients at diagnosis and 12 months time points. **(C)** Frequency of NKG2A^+^KIR^−^ and **(D)** NKG2A^−^KIR^+^ within CD56^dim^NKG2C^hi^CD57^+^ NK cells in CMV-seropositive IM and PTLD patients at diagnosis. Three CMV-seropositive IM patients were excluded in the sub-gating analysis of CD56^dim^NKG2C^hi^CD57^+^ NK cell subset due to the nearly absence of CD56^dim^NKG2C^hi^CD57^+^ NK cells. Every dot or triangle represents individual IM and PTLD patients, respectively. CMV-seropositive IM patients are highlighted as red circles. All PTLD patients were seropositive for CMV after liver transplantation. **(E)** Frequency of CD57^+^ cells within CD56^dim^ NKG2A^+^KIR^−^ NK cell subset was assessed in 13 IM and 11 PTLD patients at diagnosis to 12 months time points. Median ± interquartile range is shown. Mann-Whitney tests were applied to compare frequencies of NK cell subsets from one group or for comparisons between groups in IM and PTLD patients over time. ns, *p* > 0.05; *p ≤ 0.05; **p ≤ 0.01; ***p ≤ 0.001; and ****p ≤ 0.0001.

CMV infection is known to drive the accumulation of late-differentiated CD56^dim^NKG2C^hi^CD57^+^ NK cells (9). Expression of the receptors NKG2A and NKG2C requires covalent assembly with CD94 as a heterodimer (30) and is usually mutually exclusive on NK cells. Both NKG2A and NKG2C bind to the non-classical HLA-class I HLA-E (31) and deliver upon binding an inhibitory and activating signal, respectively. Since all PTLD patients in our cohort were CMV-seropositive, we set out to examine whether CMV skews the NK cell repertoire toward late-differentiated cells at the expense of CD56^dim^NKG2A^+^KIR^−^ NK cells. To that end, we assessed the frequency of CD56^dim^NKG2C^hi^CD57^+^ NK cells in 7 CMV-seronegative IM, 6 CMV-seropositive IM and 11 PTLD patients (Figure 5A). In agreement with previous findings (9), higher frequency of CD56^dim^NKG2C^hi^CD57^+^ NK cells was found in CMV-seropositive IM patients compared to that of CMV-seronegative IM patients, and interestingly, PTLD patients showed the highest frequency of CD56^dim^NKG2C^hi^CD57^+^ NK cells (Figure 5B). No significant change in the subset frequency over time was found in either of the CMV-seropositive patient cohorts (Figure 5B).

Subsequently, we assessed the expression of NKG2A and KIR in CD56^dim^NKG2C^hi^CD57^+^ NK cells of CMV-seropositive patients. The vast majority of CD56^dim^NKG2C^hi^CD57^+^ NK cells was NKG2A^−^KIR^+^ (Figures 5C,D).

### CD56^dim^NKG2A^+^KIR^−^CD57^+^ NK Cells Are More Frequently Found in IM Than in PTLD Patients at 12 Months Post-diagnosis

The acquisition of the terminally differentiation marker, CD57, on CD56^dim^ NKG2C^+^ NK cells upon CMV infection (9) and on CD56^dim^NKG2A^+^KIR^−^ NK cells upon EBV infection (5) has been hypothetically linked to the development of NK cell memory. Therefore, we assessed the maturation status of CD56^dim^NKG2A^+^KIR^−^ and NKG2A^−^KIR^+^ NK cell subsets using CD57 and the early-differentiation marker CD27 as control. Low but increased frequencies of CD27^+^ cells were found within CD56^dim^NKG2A^+^KIR^−^ and CD56^dim^NKG2A^−^KIR^+^ NK cell subsets in IM patients over time. Increased frequencies of CD27^+^ cells were only found within CD56^dim^NKG2A^+^KIR^−^ NK cell subset in PTLD patients over time. The frequency of CD27^+^CD56^dim^NKG2A^−^KIR^+^ NK cells was significantly lower in PTLD than IM patients at 12 months post-diagnosis (Supplementary Figure 4).

Furthermore, only few CD56^dim^NKG2A^+^KIR^−^ NK cells were CD57^+^ at the time point of diagnosis of IM and PTLD, yet, the frequency of CD57^+^CD56^dim^NKG2A^+^KIR^−^ NK cells was significantly increased in IM patients at 12 months post-diagnosis (Figure 5E). This might suggest the emergence of memory-like CD56^dim^NKG2A^+^KIR^−^CD57^+^NK cells during IM and their persistence upon remission of the disease. In contrast, the frequency of memory-like NK cells remained low in PTLD patients, consistent with the suboptimal control of EBV.

## Discussion

EBV is associated with the development of PTLD in immunosuppressed individuals such as transplant recipients. The majority of pediatric PTLD are attributable to primary EBV infection (11). With regard to the high-risk of developing PTLD in pediatric transplant recipients, routine monitoring of EBV DNA levels is recommended (32). EBV copy quantification has been used as standard practice to assess the immune control of EBV infection and determine the risk for PTLD of transplant recipients during immunosuppressive therapy. Although measurement of EBV copies in peripheral blood mononuclear cells (PBMCs) has been reported to reflect the EBV-infected circulating B cell population (27), plasma EBV viral copies have been found to be better in predicting the clinical outcomes for the management of PTLD (33). In this study, plasma EBV DNA levels were longitudinally followed as references of viral immune control in both pediatric IM and PTLD patients. Plasma EBV DNA levels were also determined in healthy donors, which served as controls.

We observed elevated plasma EBV DNA levels at the time of diagnosis in PTLD patients, which remained elevated during the longitudinal time points despite clinical remission. In contrast, plasma EBV DNA levels of IM patients dropped briskly after 1 month post-diagnosis and levels after remission were similar to those of healthy controls. However, EBV DNA levels were not predictive of the clinical outcome and persisted at high levels in pediatric liver transplant recipients irrespective of clinical recovery from PTLD. This is consistent with previous reports, as high plasma EBV DNA levels were also found in pediatric liver transplant recipients and liver transplant recipients who had recovered from PTLD, in the absence of clinical symptoms or recurrence of PTLD (25, 34, 35). This suggests that high EBV DNA levels are not necessarily predictive of clinical disease. However, we identified a weak correlation between plasma EBV and blood tacrolimus levels in our cohort of PTLD patients. In line with this observation, Guthery et al. (26) reported that immunosuppression with tacrolimus at transplantation was an independent risk factor for development of PTLD in pediatric liver transplant recipients. Thus, chronically elevated plasma EBV DNA levels after recovery from PTLD may be an indicator of suboptimal viral immune control caused by immunosuppression with tacrolimus.

Immunosuppression in transplant recipients has been recognized as a contributing factor to the suboptimal viral immune control and development of PTLD (1, 2). T cells, as the major effectors in immune control of EBV (36–38), have thus been extensively studied for their functions under the effect of immunosuppression. Suppressed T cell activation and diminished frequency of EBV-specific CD4^+^ T cells have been reported in PTLD patients compared to those of control transplant recipients (16, 17). These studies indicated that PTLD patients have an impairment of EBV-specific T cells. Previous studies have documented the complementary roles played by NK cells in viral immune control (4, 5, 10). We postulate that NK cells might also be impaired in the immunosuppressed PTLD patients contributing to the emergence of PTLD.

No significant differences in the frequency of CD56^+^ and CD56^dim^ NK cells were identified between the cohorts of IM and PTLD throughout the observation period. CD56^dim^NKG2A^+^KIR^−^ NK cells have been described to target both latent and lytic EBV infection *in vitro* (5, 10). In line with the previously published results, we found a selective accumulation of CD56^dim^NKG2A^+^KIR^−^ NK cells in IM patients. In contrast, a diminished frequency of CD56^dim^NKG2A^+^KIR^−^ NK cells was observed at diagnosis in PTLD patients that even persisted for years after recovery. As a result, late-differentiated CD56^dim^NKG2A^−^KIR^+^ NK cells formed the predominant subset in PTLD patients at all time points measured. No significant impact on the frequency of CD56^dim^NKG2A^+^KIR^−^ NK cells by the treatment of PTLD was identified. Yet, CD56^dim^NKG2A^+^KIR^−^ NK cells of PTLD patients proliferated significantly less at diagnosis and 1 month compared to those of IM patients, despite the presence of higher plasma EBV DNA levels in the former.

This difference in the frequency of NK cell subsets and the associated changes in overall NK cell function could contribute to the suboptimal EBV-specific immune control in PTLD patients. Yet, CD56^dim^NKG2A^+^KIR^−^ NK cells of PTLD and IM patients exhibited comparable cytotoxic response *ex vivo*. Therefore, we did not find any quantitative impairment in the NK cell mediated cytotoxicity in PTLD patients despite the presence of tacrolimus-mediated immunosuppression. The low frequency of CD56^dim^NKG2A^+^KIR^−^ NK cells compared to that of the IM patients could explain in part the suboptimal control of EBV viremia in PTLD patients.

Co-infection of CMV and EBV is common in immunosuppressed pediatric transplant recipients and all PTLD patients in our cohort were CMV-seropositive after transplantation. The CMV serology conversions in these patients might be caused by the CMV-positive graft they received or were already present before transplantation. According to Achour et al. (39) accumulation of CD56^dim^NKG2C^hi^CD57^+^ NK cells was found in CMV-seropositive orthotopic liver transplant recipients and the percentage of NKG2C^+^ NK cells was found to be inversely correlated with that of NKG2A^+^ NK cells. We investigated whether CMV co-infection could explain the diminished number of CD56^dim^NKG2A^+^KIR^−^ NK cells by possibly differentiating them into CD56^dim^NKG2C^+^ (NKG2A^−^) KIR^+^ NK cells in PTLD patients.

Since no CMV-seronegative liver transplant recipient was recruited in this study, we compared the frequencies of CD56^dim^NKG2C^hi^CD57^+^ NK cells in the PTLD patients to those of CMV-seronegative and -seropositive IM patients. Robust levels of the CD56^dim^NKG2C^hi^CD57^+^ NK cell subset were only found in CMV-seropositive individuals, whereby the accumulation of this subset was significantly greater in PTLD compared to CMV-seropositive IM patients (Figure 5B). In line with a mature NK cell phenotype, CD56^dim^NKG2C^hi^CD57^+^ NK cells were NKG2A^−^KIR^+^ (Figure 5D). Such CMV-associated CD56^dim^NKG2C^hi^CD57^+^ NK cells might be maintained by subclinical CMV reactivation after acute CMV infection (9). Immunosuppressed PTLD patients have repeated episodes of CMV reactivation, which might lead to more frequent differentiation of CD56^dim^NKG2A^+^KIR^−^ NK cells into CD56^dim^NKG2C^hi^CD57^+^NKG2A^−^KIR^+^ NK cells and their long-term persistence.

On the other hand, the expression of the terminal differentiation marker CD57 on CD56^dim^NKG2C^hi^ NK cells has been proposed to indicate memory-like properties after acute CMV infection (9, 40). The significant increase in the frequency of CD57^+^CD56^dim^NKG2A^+^KIR^−^ NK cells in IM patients at 12 months post-diagnosis could indicate the presence of memory-like CD56^dim^NKG2A^+^KIR^−^ NK cells, which may contribute to the long-term control of EBV. Together, the data suggest that CMV infection in immunosuppressed PTLD patients drives NK cells to terminally differentiated NKG2C^hi^CD57^+^NKG2A^−^KIR^+^ NK cell subset as described in other settings and might impair the generation and maintenance of EBV-reactive CD56^dim^NKG2A^+^KIR^−^ NK cells. Indeed, CMV disease is a putative risk factor for the development of PTLD in liver transplant recipient (41).

Tacrolimus has been reported to increase the viral burden of EBV in infected humanized mice, leading to more frequent tumor formation (42). Tacrolimus impairs NK cell proliferation in a dose-dependent manner and selectively down-regulates the expression of NKG2D, CD48 and DNAM1 on NK cells (43). Although the effect of tacrolimus on the expression of NKG2A has not been investigated in the study by Kim et al., these results suggest that the immunosuppressive treatment could directly contribute to the relative reduction of CD56^dim^NKG2A^+^KIR^−^ NK cells in PTLD patients, however, no statistically significant difference could be identified in our current study. Further research with a larger study size is needed to investigate the direct or indirect effects of tacrolimus on the CD56^dim^NKG2A^+^KIR^−^ NK cell subset.

To conclude, we have comprehensively examined the phenotype and function of distinct NK cell subsets against EBV-infected B cells in PTLD patients under immunosuppression during a longitudinal follow-up study. By comparison with the EBV-associated benign lymphoproliferative disorder IM, we demonstrate that co-infection of CMV and EBV diminishes the frequency of CD56^dim^NKG2A^+^KIR^−^ NK cells and may contribute to suboptimal immune control of EBV in immunosuppressed children with PTLD.

## Data Availability Statement

The datasets generated for this study are available on request to the corresponding author.

## Ethics Statement

The studies involving human participants were reviewed and approved by Institutional Review Board of the University of Hong Kong. Written informed consent to participate in this study was provided by the participants' legal guardian/next of kin.

## Author Contributions

JL, TA, and AW performed experiments. JL, TA, KH, DM, NC, CM, and AC analyzed and interpreted data. JL, DM, and NC performed statistical analysis. KC performed the serological assays. JL, TA, DM, NC, CM, and AC wrote the manuscript. KH, CM, and AC designed the research. AC coordinated the project, recruited, and followed all patients. All authors reviewed the manuscript.

## Conflict of Interest

The authors declare that the research was conducted in the absence of any commercial or financial relationships that could be construed as a potential conflict of interest.
