# Supplementary material for: Co-infection of Cytomegalovirus and Epstein-Barr Virus Diminishes the Frequency of CD56dimNKG2A+KIR− NK Cells and Contributes to Suboptimal Control of EBV in Immunosuppressed Children With Post-transplant Lymphoproliferative Disorder
Source: Front Immunol. 2020 Jun 17;11:1231. doi: 10.3389/fimmu.2020.01231 (PMC7311655; doi:10.3389/fimmu.2020.01231)
Supplement: Supplementary file 1 [file Data_Sheet_1.PDF]

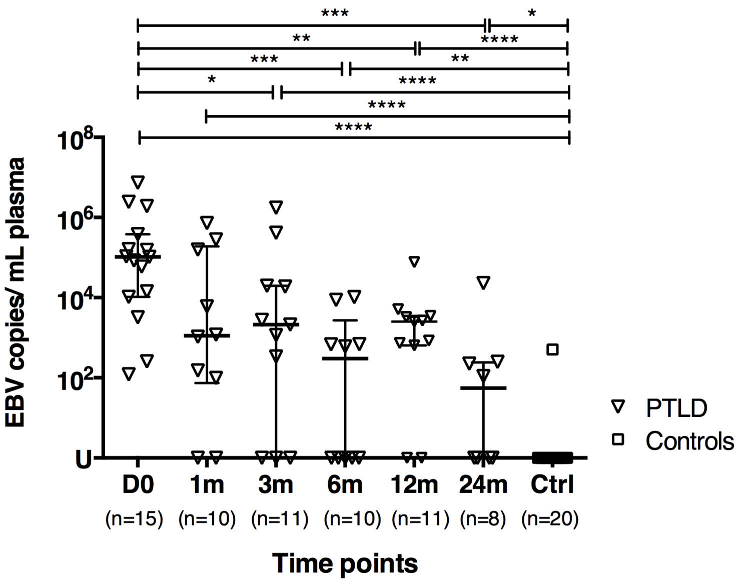

Supplementary Figure 1. PTLD patients exhibit persistent elevated plasma EBV DNA levels up to 24 months after diagnosis. Plasma EBV DNA levels were quantified in healthy controls and PTLD patients at diagnosis, as well as during recovery at 1, 3, 6 and 12 months. Additional plasma EBV DNA levels were also quantified in PTLD patients at 24 months after diagnosis. EBV DNA levels were assessed by qPCR and reported as EBV DNA copy numbers per ml plasma. Median ± interquartile range is shown. Mann-Whitney tests were applied to compare plasma EBV DNA levels at diagnosis to other time points within one cohort or for comparison between different cohorts of study subjects. U, undetectable level; Ctrl, healthy controls; ns, p-value > 0.05; *, p-value ≤ 0.05; **, p-value ≤ 0.01; ***, p-value ≤ 0.001; ****, p-value ≤ 0.0001.
