# Supplementary material for: Co-infection of Cytomegalovirus and Epstein-Barr Virus Diminishes the Frequency of CD56dimNKG2A+KIR− NK Cells and Contributes to Suboptimal Control of EBV in Immunosuppressed Children With Post-transplant Lymphoproliferative Disorder
Source: Front Immunol. 2020 Jun 17;11:1231. doi: 10.3389/fimmu.2020.01231 (PMC7311655; doi:10.3389/fimmu.2020.01231)
Supplement: Supplementary file 2 [file Data_Sheet_2.PDF]

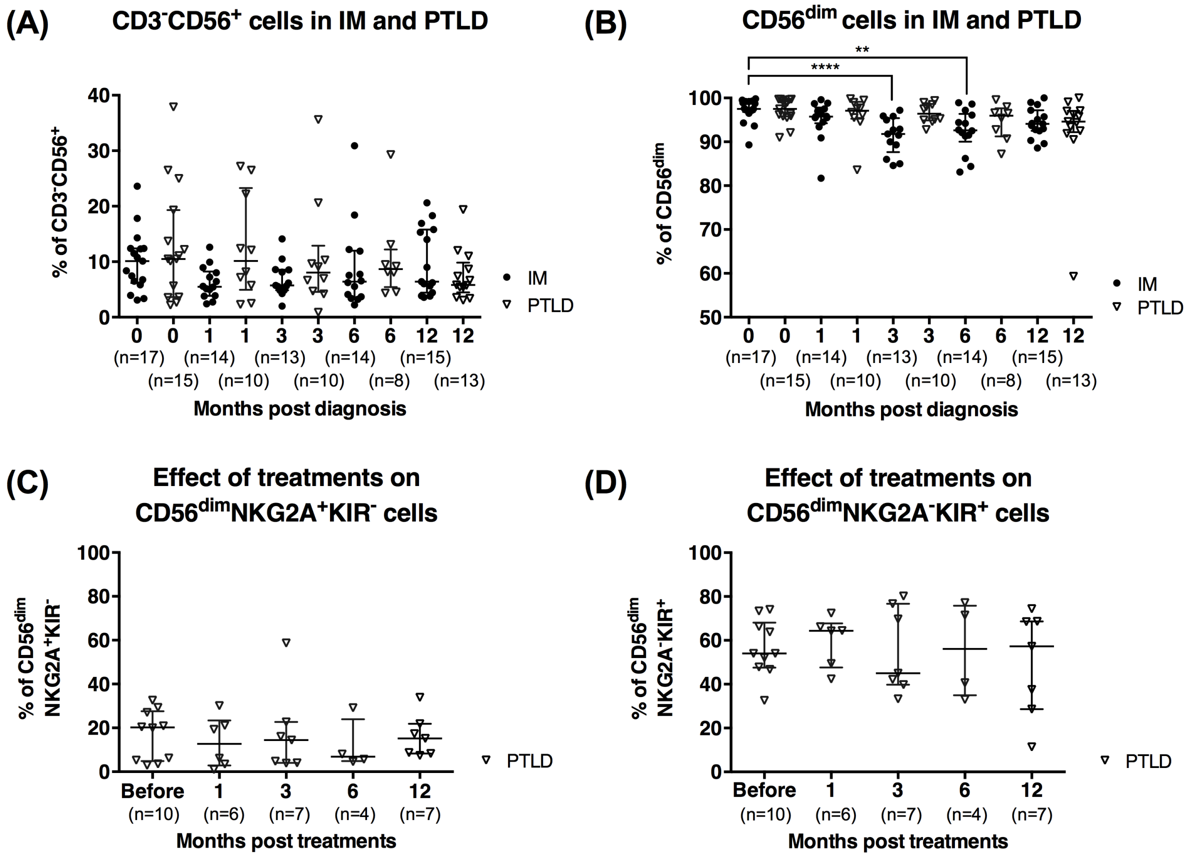

Supplementary Figure 2. No significant differences in the frequencies of CD3$^-$CD56$^+$, CD56^{dim}, CD56^{dim}NKG2A$^+$KIR$^-$ and CD56^{dim}NKG2A$^-$KIR$^+$ NK cells over time or treatment effect. Longitudinal changes in **(A)** CD3$^-$CD56$^+$ and **(B)** CD56^{dim} NK cell frequencies were monitored from diagnosis to 12 months recovery time points in 17 IM and 15 PTLD patients, respectively. Frequencies of **(C)** CD56^{dim}NKG2A$^+$KIR$^-$ and **(D)** CD56^{dim}NKG2A$^-$KIR$^+$ NK cells were monitored at diagnosis (before treatments) and time point 1, 3, 6 and 12 months after treatments in 10 PTLD patients. Median ± interquartile range is shown. Mann-Whitney tests were applied to compare the frequencies of NK cells within one cohort or for comparison of both cohorts. **, p-value ≤ 0.01; ****, p-value ≤ 0.0001.
