# Supplementary material for: Co-infection of Cytomegalovirus and Epstein-Barr Virus Diminishes the Frequency of CD56dimNKG2A+KIR− NK Cells and Contributes to Suboptimal Control of EBV in Immunosuppressed Children With Post-transplant Lymphoproliferative Disorder
Source: Front Immunol. 2020 Jun 17;11:1231. doi: 10.3389/fimmu.2020.01231 (PMC7311655; doi:10.3389/fimmu.2020.01231)
Supplement: Supplementary file 3 [file Data_Sheet_3.PDF]

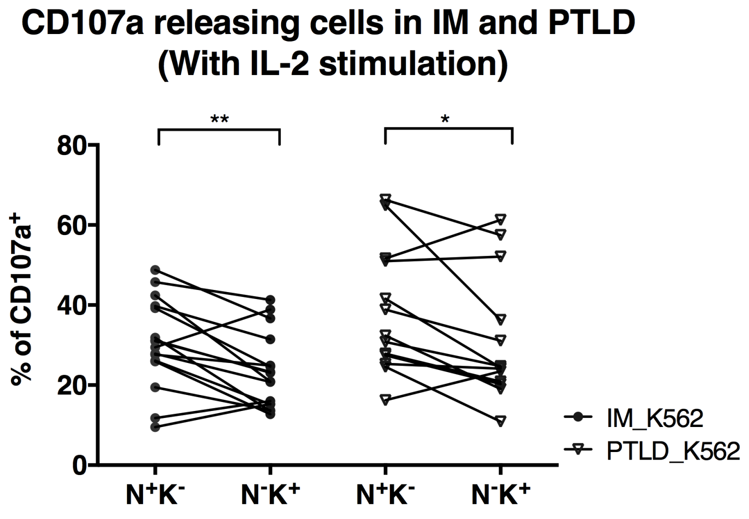

Supplementary Figure 3. CD56^{dim}NKG2A^{+}KIR^{-} NK cells of both PTLD and IM patients exhibited comparable cytotoxic degranulation in response to erythroleukemia K562 cell line. PBMCs from 16 IM patients at the 12 months recovery time point and PBMCs from 13 PTLD patients at the 24 months recovery time point were stimulated with IL-2 overnight prior to co-culture with the erythroleukemia K562 cell line on the next day at effector to target ratio of 10:1 for 6 hours. Frequencies of cells with CD107a surface expression in the CD56^{dim}NKG2A^{+}KIR^{-} and CD56^{dim}NKG2A^{-}KIR^{+} NK cell subsets in 16 IM patients and 13 PTLD patients were assessed by flow cytometry at the end of the co-culture. Every single line represents 1 individual. Wilcoxon matched-pairs signed rank tests were applied to compare data from one group. *, p-value ≤0.05; **, p-value ≤ 0.01.
