# Supplementary material for: Co-infection of Cytomegalovirus and Epstein-Barr Virus Diminishes the Frequency of CD56dimNKG2A+KIR− NK Cells and Contributes to Suboptimal Control of EBV in Immunosuppressed Children With Post-transplant Lymphoproliferative Disorder
Source: Front Immunol. 2020 Jun 17;11:1231. doi: 10.3389/fimmu.2020.01231 (PMC7311655; doi:10.3389/fimmu.2020.01231)
Supplement: Supplementary file 4 [file Data_Sheet_4.PDF]

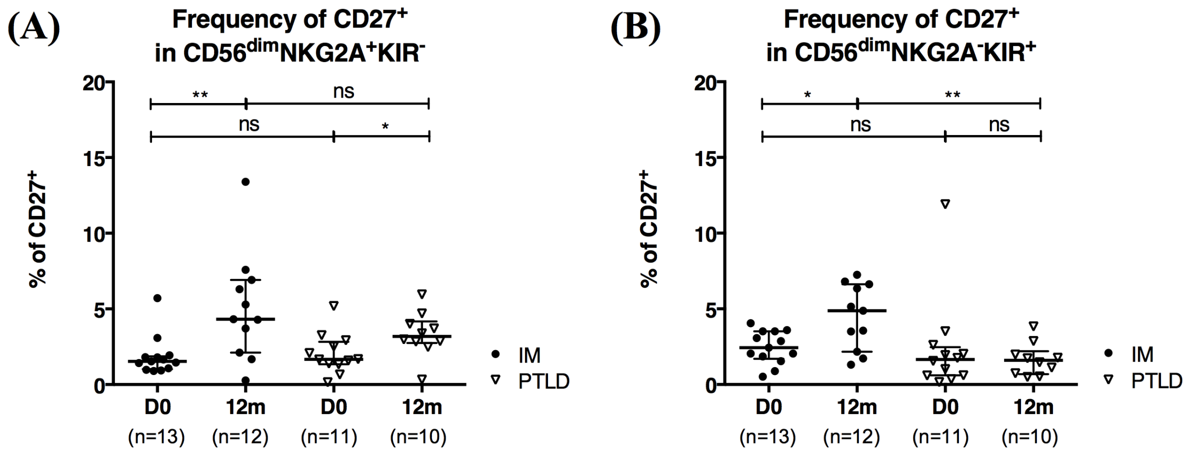

Supplementary Figure 4. Low but increased frequency of CD27^{+} cells was found within CD56^{dim} NKG2A^{+}KIR^{-} NK cell subset in IM and PTLD patients over time. Frequencies of CD27^{+} cells within **(A)** CD56^{dim} NKG2A^{+}KIR^{-} and **(B)** CD56^{dim} NKG2A^{-}KIR^{+} NK cell subsets were assessed in 13 IM and 11 PTLD patients at diagnosis to 12 months post-diagnosis time points. Median ± interquartile range is shown. Mann-Whitney tests were applied to compare the frequencies of CD27^{+} cells within one cohort or for comparison of both cohorts. *, p-value ≤ 0.05; **, p-value ≤ 0.01.
