# Supplementary material for: Co-infection of Cytomegalovirus and Epstein-Barr Virus Diminishes the Frequency of CD56dimNKG2A+KIR− NK Cells and Contributes to Suboptimal Control of EBV in Immunosuppressed Children With Post-transplant Lymphoproliferative Disorder
Source: Front Immunol. 2020 Jun 17;11:1231. doi: 10.3389/fimmu.2020.01231 (PMC7311655; doi:10.3389/fimmu.2020.01231)
Supplement: Supplementary file 6 [file Data_Sheet_5.PDF]

Supplementary Table 2. Mean and standard deviation of time points. The mean of time point and standard deviation between the proposed and the actual time point selected in 17 IM and 15 PTLD patients.

Patient cohort	Proposed time point labeled in figures	Mean (months)	SD	N
IM	Diagnosis	0	0	17
	1 month	0.80	0.305	15
	3 months	2.88	0.433	12
	6 months	6	0	13
	12 months	11.88	0.806	16
PTLD	Diagnosis	0	0	15
	1 month	1.09	0.563	10
	3 months	2.97	0.386	12
	6 months	5.88	0.354	8
	12 months	12.73	2.728	13
	24 months	24.17	3.433	12
